# Cold atmospheric plasma enhances osteoblast differentiation

**DOI:** 10.1371/journal.pone.0180507

**Published:** 2017-07-06

**Authors:** Kanako Tominami, Hiroyasu Kanetaka, Shota Sasaki, Takayuki Mokudai, Toshiro Kaneko, Yoshimi Niwano

**Affiliations:** 1 Graduate School of Biomedical Engineering, Tohoku University, Sendai, Japan; 2 Graduate School of Dentistry, Tohoku University, Sendai, Japan; 3 Department of Electronic Engineering, Tohoku University, Sendai, Japan; Charles P. Darby Children's Research Institute, UNITED STATES

## Abstract

This study was designed to assess the effects of cold atmospheric plasma on osteoblastic differentiation in pre-osteoblastic MC3T3-E1 cells. Plasma was irradiated directly to a culture medium containing plated cells for 5 s or 10 s. Alkaline phosphatase (ALP) activity assay and alizarin red staining were applied to assess osteoblastic differentiation. The plasma-generated radicals were detected directly using an electron spin resonance-spin trapping technique. Results show that plasma irradiation under specific conditions increased ALP activity and enhanced mineralization, and demonstrated that the yield of radicals was increased in an irradiation-time-dependent manner. Appropriate plasma irradiation stimulated the osteoblastic differentiation of the cells. This process offers the potential of promoting bone regeneration.

## Introduction

In a modern society with a large aging population, age-related diseases such as decubitus ulcers and periodontitis caused by a weakened immune system have come to present an important social problem. For instance, periodontitis, an inflammatory disease that causes progressive destruction of tooth-supporting tissues including alveolar bone, root cementum, and periodontal ligaments, affects a large share of humanity worldwide. Periodontitis destroys tooth-supporting tissues and eventually causes tooth loss. Reconstituting lost periodontal structures persists as a difficult medical challenge [1, 2].

Important treatment-related points for these chronic infectious diseases are the sterilization of surrounding tissues and the enhancement of new tissue formation, which both promote wound healing of bone defects and their surrounding tissues [3]. For example, augmentation is necessary in many cases before dental-implant implantation because periodontal disease tends to absorb the alveolar ridge. Nevertheless, alveolar ridge augmentation with periodontal disease is not a simple surgical procedure.

Bone remodeling processes are controlled by a balance between bone formation and bone resorption. Osteoblasts are crucially important for bone remodeling processes. Satisfactory bone regeneration is especially necessary for healing critical-size bone defects after skeletal injuries in orthopedic surgery and dentistry [4].

In recent years, as an emerging field, plasma medicine has been widely developed for various applications such as medical equipment sterilization [5, 6], gene transfection [7], cell proliferation [8], and wound healing [9]. These non-thermal plasmas support the direct treatment of biological systems without the thermal damage that can occur when using conventional thermal plasma. Consequently, because it has both bactericidal action and bone regeneration effects, atmospheric plasma might provide a means for resolving difficulties related to treating chronic infectious diseases such as decubitus ulcers and periodontitis.

This study investigated the effects of cold atmospheric plasma on a pre-osteoblastic cell line: MC3T3-E1. We elucidated the effects of plasma irradiation on proliferation rates, alkaline phosphatase (ALP) activity and alizarin red staining (as a marker of osteoblast maturation). The MC3T3-E1 cells represent a suitable model for studying osteogenic development *in vitro* [10].

Depending on the discharge conditions, an electrical discharge such as plasma can be an effective source of electrons, ions, free radicals, and UV light. Reportedly, cold atmospheric plasma can induce apoptosis through short-lived reactive chemicals such as reactive oxygen species (ROS) and UV radiation [11, 12]. Moreover, physical stresses such as electrical stimulation and through mechanical loading reportedly promote bone regeneration and healing of bone fractures [3, 13].

Therefore, we also determined the level of radicals in a solution irradiated with plasma. Specifically, this study presents electron spin resonance (ESR) determination of three types of radicals generated in a plasma–water system: hydroxyl radical (^•^OH), hydrogen radical (^•^H), and superoxide anion radical (^•^O_2_^-^).

## Materials and methods

### 1. Plasma irradiation

**Fig 1:**
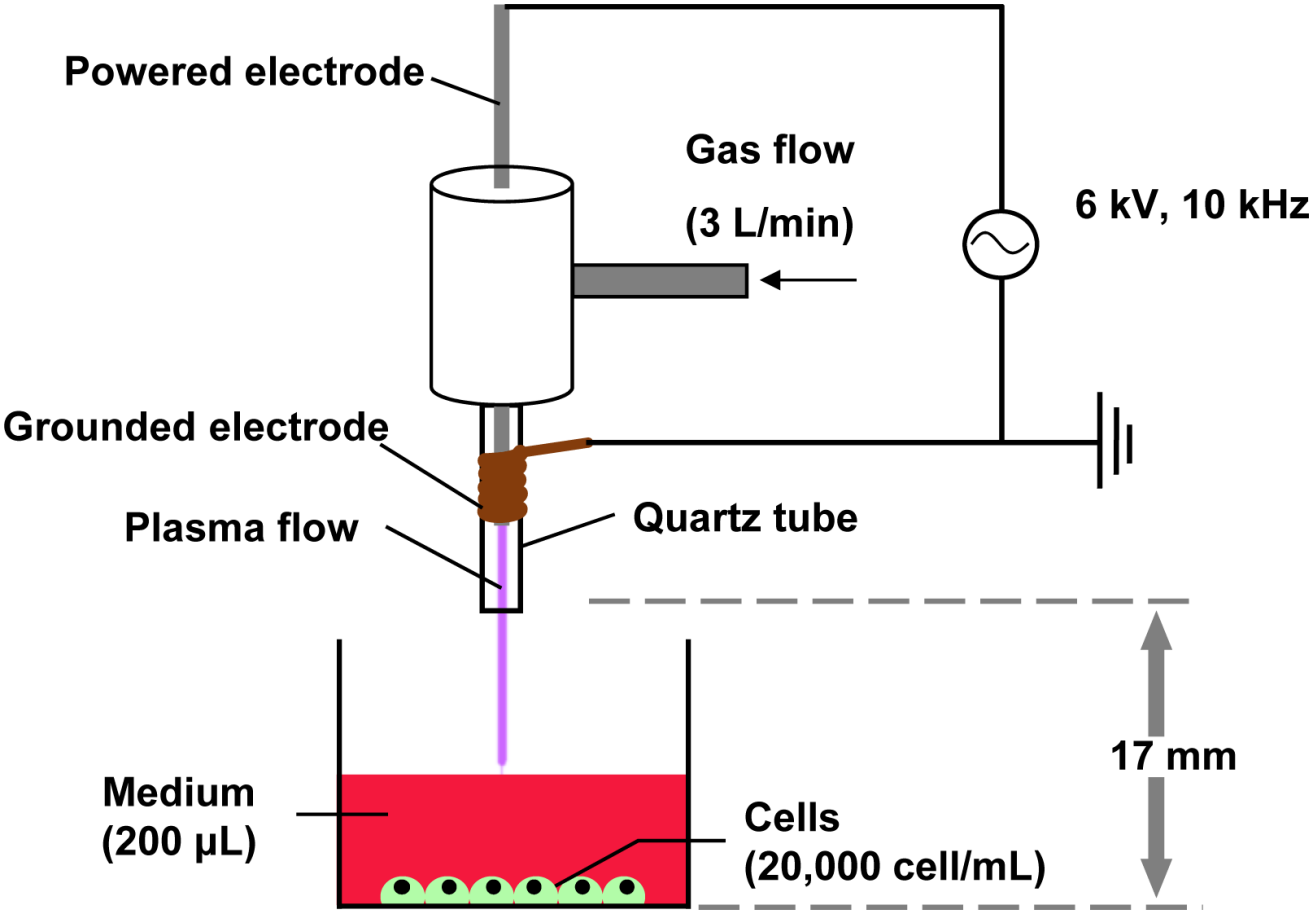
Schematic illustration of the experimental setup used for this study. Low-temperature atmospheric-pressure plasma was irradiated directly to cells in 200 μL of medium in the 24-well plate for 5 s and 10 s. The He gas flow rate used for the experiments was 3 L/min.

Cold atmospheric plasma produced using an experimental setup similar to that described previously [14] is shown schematically in Fig 1. The plasma jet apparatus comprises a dielectric quartz tube (6 mm inner diameter, 10 mm outer diameter) with two conducting electrodes. One electrode, a 1.5 mm-diameter tungsten rod, is placed inside the tube along the tubal axis, ending about 30 mm before the nozzle exit of the tube. The second electrode, a metal coil (copper), is attached to the outer wall of the tube near the nozzle exit. The distance from the nozzle to the culture medium is 16.2 mm. When helium (He) gas flows (3 L/min) through the quartz tube, cold atmospheric plasma is discharged by high voltage applied between the two electrodes. The discharge voltage and operating frequency are maintained, respectively, at 6 kV and 10 kHz. The plasma is irradiated directly to each solution in a well of a 24-well plate for each assay.

### 2. Cell culture and the induction of differentiation

The MC3T3-E1 murine pre-osteoblast cell line was grown at 37°C in an atmosphere of 5% CO_2_/95% air in Dulbecco’s modified Eagle’s medium (DMEM; Wako Pure Chemical Industries Ltd., Osaka, Japan) supplemented with 10% (v/v) fetal bovine serum (FBS; Invitrogen Corp., Carlsbad, CA, USA), 100 U/mL penicillin (Meiji-Seika Kaisha Ltd., Tokyo, Japan), and 100 μg/mL streptomycin (Meiji-Seika Kaisha Ltd., Tokyo, Japan).

Cells suspended in DMEM with 10% (v/v) FBS and penicillin/streptomycin (PS) were seeded at a density of 2 × 10^4^ cells/well in 24-well culture plates, and were incubated for 2 days at 37°C with 5% CO_2_. To differentiate the cells, the medium was replaced with DMEM with 10% (v/v) FBS and an osteoblast-inducer reagent (OIR; Takara Bio Inc., Otsu, Japan), which included L-ascorbic acid, hydrocortisone, and β-glycerophosphate.

The cells in the 24-well plate were treated directly with plasma in the presence of 200 μL medium for differentiation to prevent the cells from drying. Direct plasma irradiation of cells was conducted once a day for five consecutive days at a distance of 17 mm from the adherent cells of each well in open air. Bone morphogenetic protein 2 (BMP2; Peprotech Inc., Rocky Hill, NJ, USA) was used as a positive control at a concentration of 100 ng/ml. Cells without plasma irradiation were used as a negative control.

### 3. Alkaline phosphatase (ALP) activity assays

Direct plasma irradiation of cells cultured with DMEM with 10% FBS and OIR for differentiation was conducted once a day for five consecutive days. The plasma was irradiated for 5 s or 10 s each time. Cells were cultured for 7 days when irradiated with plasma five times, or for 14 days when irradiated with plasma 10 times. After 7 or 14 days for cell differentiation with or without plasma irradiation, the cells were rinsed three times with phosphate buffered saline (PBS), and 200 μL of alkaline phosphate yellow (pNPP) liquid substrate (Sigma-Aldrich Corp., USA). In addition, 2 μL of 10% Triton-X/PBS was added to each well, with subsequent incubation at 37°C for 15 min. After the yellow color appeared, the reaction was terminated with 75 μL of 2 M NaOH. Then absorbance at 450 nm was read using a microplate spectrometer (GloMax-Multi Detection System; Promega Corp.). This assay was conducted in triplicate.

### 4. Influence of hydrogen peroxide (H_2_O_2_) concentrations on ALP activity

The concentration of H_2_O_2_ in pure water, generated by plasma irradiation, was ascertained using a colorimetric method [15] based on peroxide-mediated oxidation of Fe^2+^ followed by reaction of Fe^3+^ with xylenol orange (XO). This ferrous oxidation—xylenol orange (FXO) assay detects hydroperoxides [16, 17].

At each time point, 400 μL of the pure water with plasma irradiation was added immediately to 400 μL of the XO mixture (containing 0.5 mM ferrous ammonium sulfate hexahydrate (Fe(NH_4_)_2_(SO_4_)_2_), 12.5 mM H_2_SO_4_, 0.2 mM xylenol orange tetra sodium salt, 200 mM D-sorbitol). Samples were kept at room temperature for 45 min. The absorbance at 560 nm was read using a microplate spectrometer (GloMax-Multi Detection System; Promega Corp., Madison, WI, USA). The total peroxide concentration was determined using a standard curve prepared with different concentrations of H_2_O_2_ in pure water.

To evaluate the influence of the H_2_O_2_ on the ALP activity of MC3T3-E1, cells were incubated for 14 days under several H_2_O_2_ concentrations varied in the range of 1.5× 10^−6^ M to 6.0 × 10^−6^ M.

### 5. Cell proliferation and DNA quantification

To evaluate the influence of plasma irradiation on the cell proliferation of MC3T3-E1, DNA quantification of the cells was performed for each condition. Direct plasma irradiation of cells cultured with DMEM with 10% FBS and OIR for differentiation was conducted once a day for five consecutive days. The plasma was irradiated for 5 s or 10 s each time. Cells were cultured for 14 days during which plasma was irradiated 10 times. After 14 days of cell differentiation with or without plasma irradiation, DNA was isolated with the AllPrep DNA/RNA/Protein Mini Kit (Qiagen Inc., Valencia, CA, USA) and QIA shredder (Qiagen Inc.) according to the manufacturer’s protocol. Cells were harvested using a cell scraper (Sumitomo Bakelite Co. Ltd., Tokyo, Japan). Following extraction, the DNA was quantified by absorbance at 260 nm using a spectrophotometer (e-Spect ES-2; Malcom Co., Ltd., Tokyo, Japan).

### 6. Alizarin red staining

Direct plasma irradiation of cells cultured with DMEM with 10% FBS and OIR for differentiation was conducted once a day for five consecutive days. The plasma was irradiated for 5 s or 10 s at each time. Cells were cultured for 21 days. Plasma irradiation of cells was conducted during the first week so that the number of plasma irradiations became five or the first two weeks with 10 times plasma irradiation.

After 21 days of differentiation, the cells were washed three times with PBS and fixed in 10% neutral buffered formalin for 20 min at 4°C. Then they were rinsed three times with pure water. These cells were stained with 1% (w/v) alizarin red S, pH 6.3–6.4, for 5 min with gentle agitation at room temperature. The cells were rinsed three times with pure water. Alizarin red was extracted from fixed cells with 200 μL of 10% (w/v) cetylpyridinium chloride (CPC) for 30 min with gentle agitation. Absorbance of the extracted alizarin red in CPC solution was read at 560 nm using a microplate spectrometer (GloMax-Multi Detection System; Promega Corp.).

### 7. RNA extraction and quantitative RT-PCR analysis

The respective expressions of ALP and OCN mRNA were analyzed under the same conditions as the expriments "ALP activity assays" and "Alizarin red staining". After the cells were washed with PBS, total RNA was extracted using QIA shredder and RNeasy Mini Kit (Qiagen Inc., Hilden, Germany) according to the manufacturer’s instructions. The RNA content was measured spectrophotometrically at 260 nm. For quantitative RT-PCR analysis, the following synthetic oligonucleotide primers were used (Table 1): for ALP, forward 5′-TACGCTCACAACAACTACCA and reverse 5′-GGGAATGTAGTTCTGCTCAT; for OCN, forward 5′-CTCTCTCTGCTCACTCTGCT and reverse 5′-CGGAGTCTGTTCACTACCTTA. β-Actin housekeeping gene was amplified with forward 5′- TGGCACCCAGCACAATGAA and reverse 5′- CTAAGTCATAGTCCGCCTAGAAGCA. Total RNA (1 μg) was reverse-transcribed into cDNA using ReverTra Ace qPCR RT kit (Toyobo Co. Ltd., Osaka, Japan) according to the manufacturer’s instructions. Quantitative PCR reactions were then performed using Thunderbird SYBR qPCR Mix (Toyobo Co. Ltd.). PCR cycling conditions using a Thermal Cycler Dice Real time System (TP800; Takara Bio Inc., Otsu, Japan) were the following: initial denaturation at 95°C for 1 min, followed by 50 cycles of denaturation at 95°C for 5 s, annealing at 60°C for 30 s, and extension at 72°C for 30 s. Dice Real Time System software (version 2.10B; Takara Bio Inc.) was used to extract and analyze the PCR data. The copy numbers of genes measured in triplicate were normalized against the average copy number of β-actin.

**Table 1:** Primer sequence for real-time PCR.

Gene	Primers (5′–3′)	
	Forward	Reverse
***Alp***	tacgctcacaacaactacca	gggaatgtagttctgctcat
***OCN***	ctctctctgctcactctgct	cggagtctgttcactacctta
***β-actin***	tggcacccagcacaatgaa	ctaagtcatagtccgcctagaagca

### 8. Electron spin resonance (ESR) analysis

An ESR-spin trapping technique using 5,5-dimethyl-1-pyrroline-*N*-oxide (DMPO; Labtech International Ltd., Tokyo, Japan) or 5-(2,2-dimethyl-1,3-propoxy cyclophosphoryl)-5-methyl-pyrroline *N*-oxide (CYPMPO; Mikuni Pharmaceutical Industrial Co. Ltd., Osaka, Japan) was applied to ascertain free radicals such as ^•^OH, ^•^H, and ^•^O_2_^-^ [18]. Actually, CYPMPO performs better than DMPO for ^•^O_2_^-^ measurements because its spin adduct has a longer lifetime than that of DMPO-OOH, a spin adduct of ^•^O_2_^-^ and DMPO [19, 20]. Therefore, we used CYPMPO as the spin-trapping agent for ^•^O_2_^-^.

The ^•^OH generated by the He plasma irradiation of pure water for 10 s was measured. An aliquot (1 mL) of each liquid sample containing 300 mM DMPO was used.

In addition to investigation of the actual effect of ^•^O_2_^-^ produced in pure water by the He plasma on osteoblast differentiation, a volume of 200 μl liquid was used. CYPMPO was added to the liquid and was mixed thoroughly (final concentration: 10 mM) before plasma irradiation for 10 s.

The measurement conditions for ESR (X-band ESR spectrometer; FES-FA100, JEOL, Tokyo, Japan) were the following: 4 mW microwave power; 9.43 GHz microwave frequency; magnetic field, 337.108 mT for DMPO, 337.071 mT for CYPMPO; ±5 mT field sweep width; 100 kHz field modulation; 0.1 mT modulation width; 0.03 s time constant; and 60 s sweep time.

A given concentration of 4-hydroxy-2,2,6,6-tetramethylpiperidine 1-oxyl (Sigma-Aldrich Corp.) was used as a standard to calculate the DMPO-OH concentration (an adduct of DMPO and ^•^OH). The ESR spectrum of manganese held in the ESR cavity was used as an internal standard. Each assay was done in triplicate.

### 9. Statistical analysis

All data represent the mean ± standard deviation (SD). Significant differences between groups were assessed using one-way analysis of variance, followed by the Tukey–Kramer multiple comparison test. *P-*values < 0.05 were inferred as significant.

## Results

### 1. Influence of plasma on ALP activity

**Fig 2:**
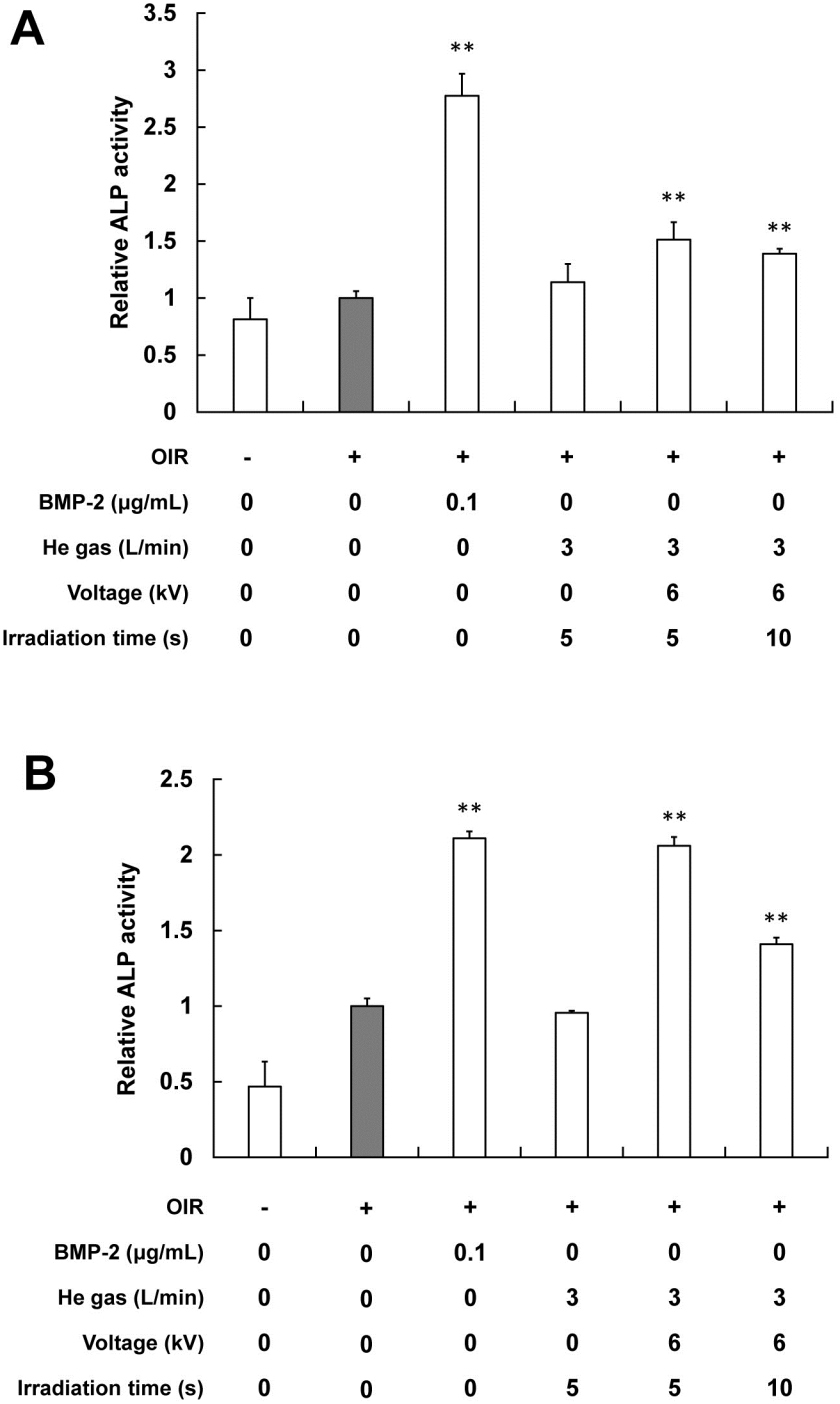
Effect of the plasma irradiation on the ALP activity of MC3T3-E1. Cells were incubated for 7 days (A) or 14 days (B). The ALP activity is expressed as a relative value for which the value of the negative control (gray) is set to 1. Each value is the mean with SD (*n* = 5). ** *p*< 0.01 vs. control.

The ALP activity in the plasma-treated MC3T3-E1 cells was determined after 7 and 14 days of differentiation (Fig 2A and 2B). The positive control (BMP-2) increased ALP activity on both days. The He plasma increased the ALP activity of the MC3T3-E1 cells on both days. The activity was higher by irradiation for 5 s/day than for 10 s/day. The greater the number of irradiations, the higher the activity became, given equal irradiation periods.

### 2. Influence of H_2_O_2_ concentrations on ALP activity

**Fig 3:**
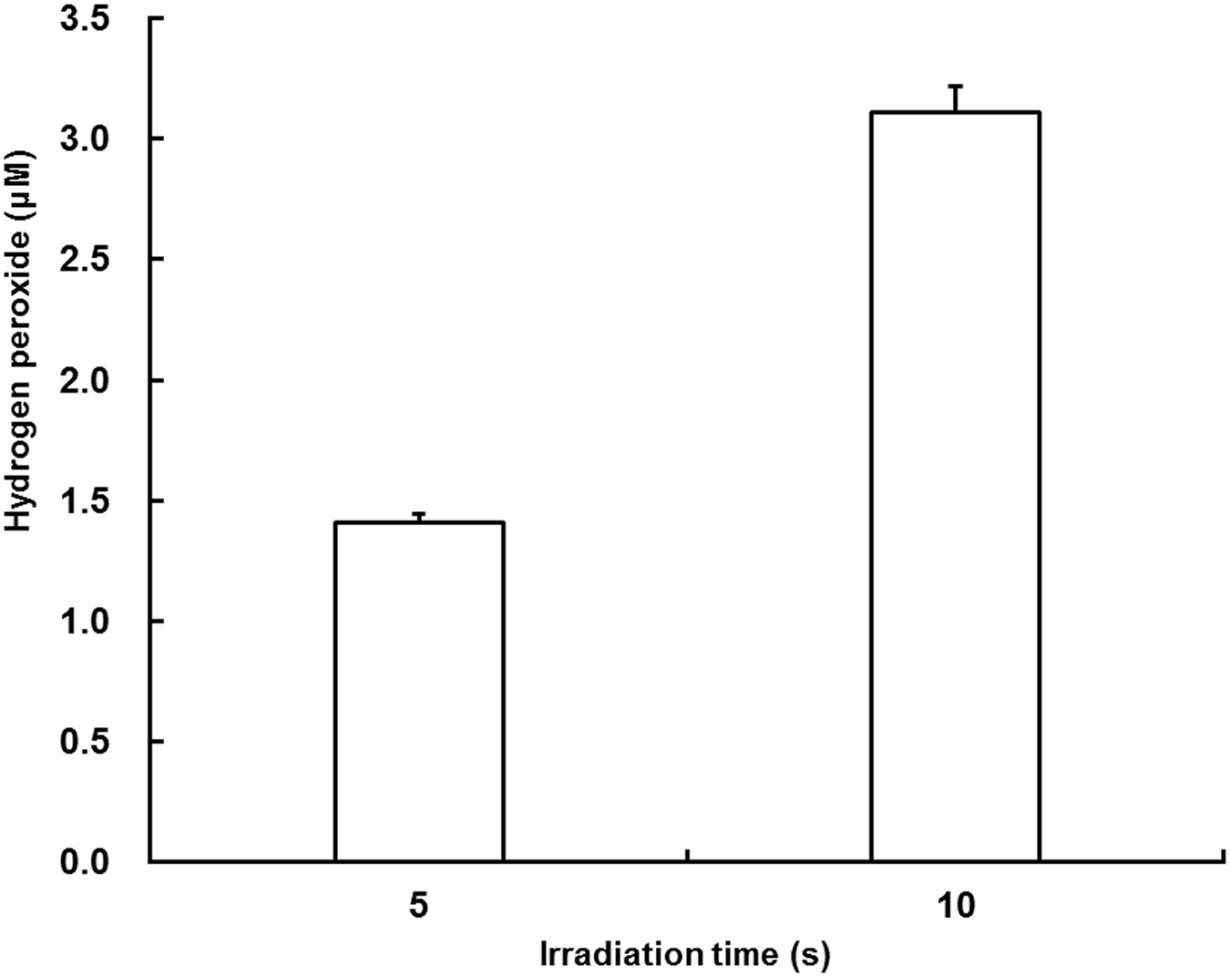
Concentration of hydrogen peroxide generated by the plasma. Each value is the mean with SD (*n* = 3).

Fig 3 presents results of H_2_O_2_ concentrations in plasma-treated pure water. The H_2_O_2_ concentrations increased with irradiation time. Irradiation for 10 s produced a yield of approximately 3 μM H_2_O_2_.

**Fig 4:**
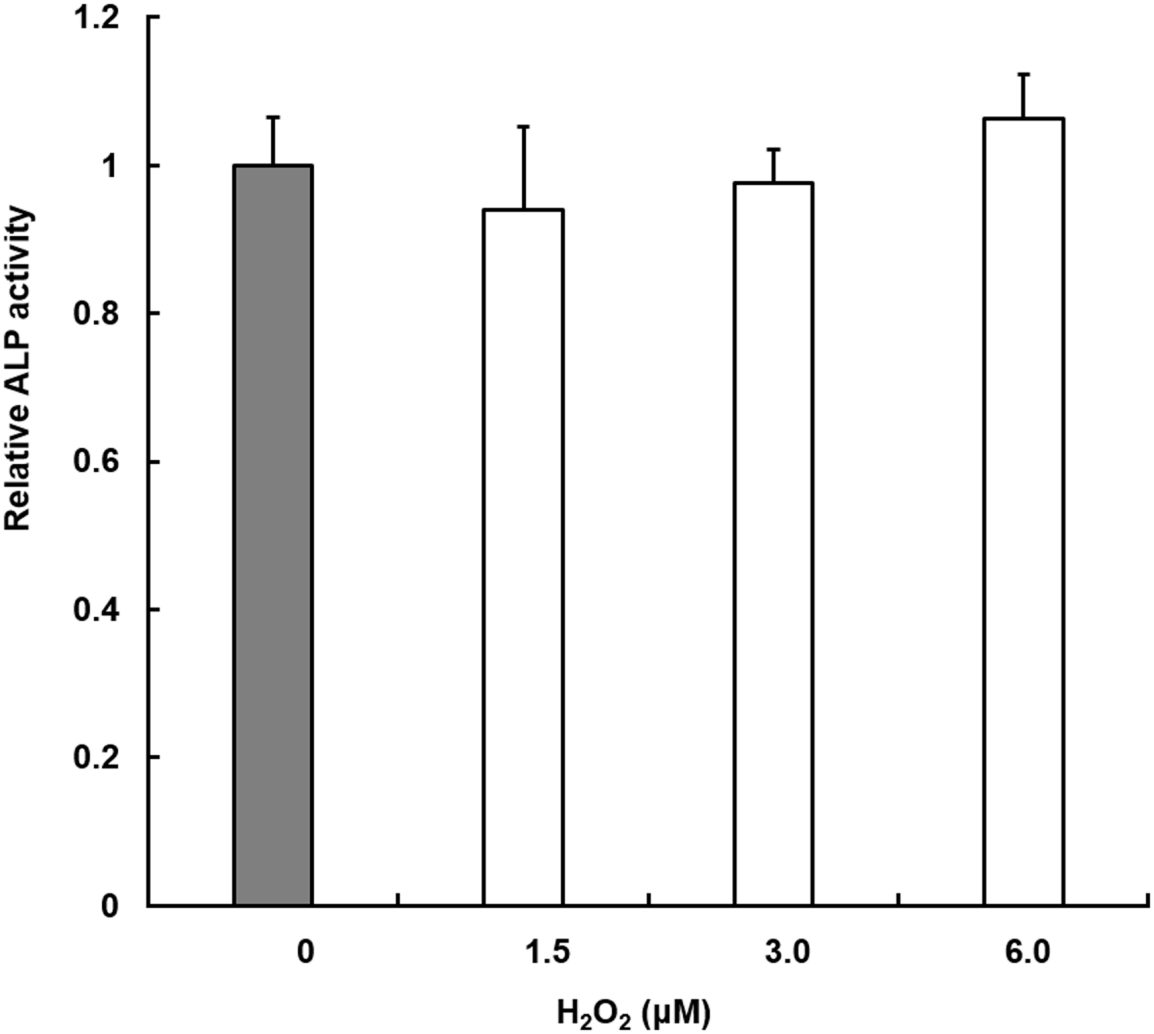
Effect of the H_2_O_2_ on the ALP activity of MC3T3-E1. Cells were incubated for 14 days. The ALP activity is expressed as a relative value for which the value of the negative control (gray) is set to 1. Each value is the mean with SD (*n* = 5). **p < 0.01 vs. control.

The hydrogen peroxide concentration was varied in a range of 1.5 × 10^−6^ M– 6.0 × 10^−6^ M. Cells incubated with OIR for 14 days without hydrogen peroxide were used as controls. No significant difference was found between the groups (Fig 4).

### 3. Influence of plasma on cell proliferation

**Fig 5:**
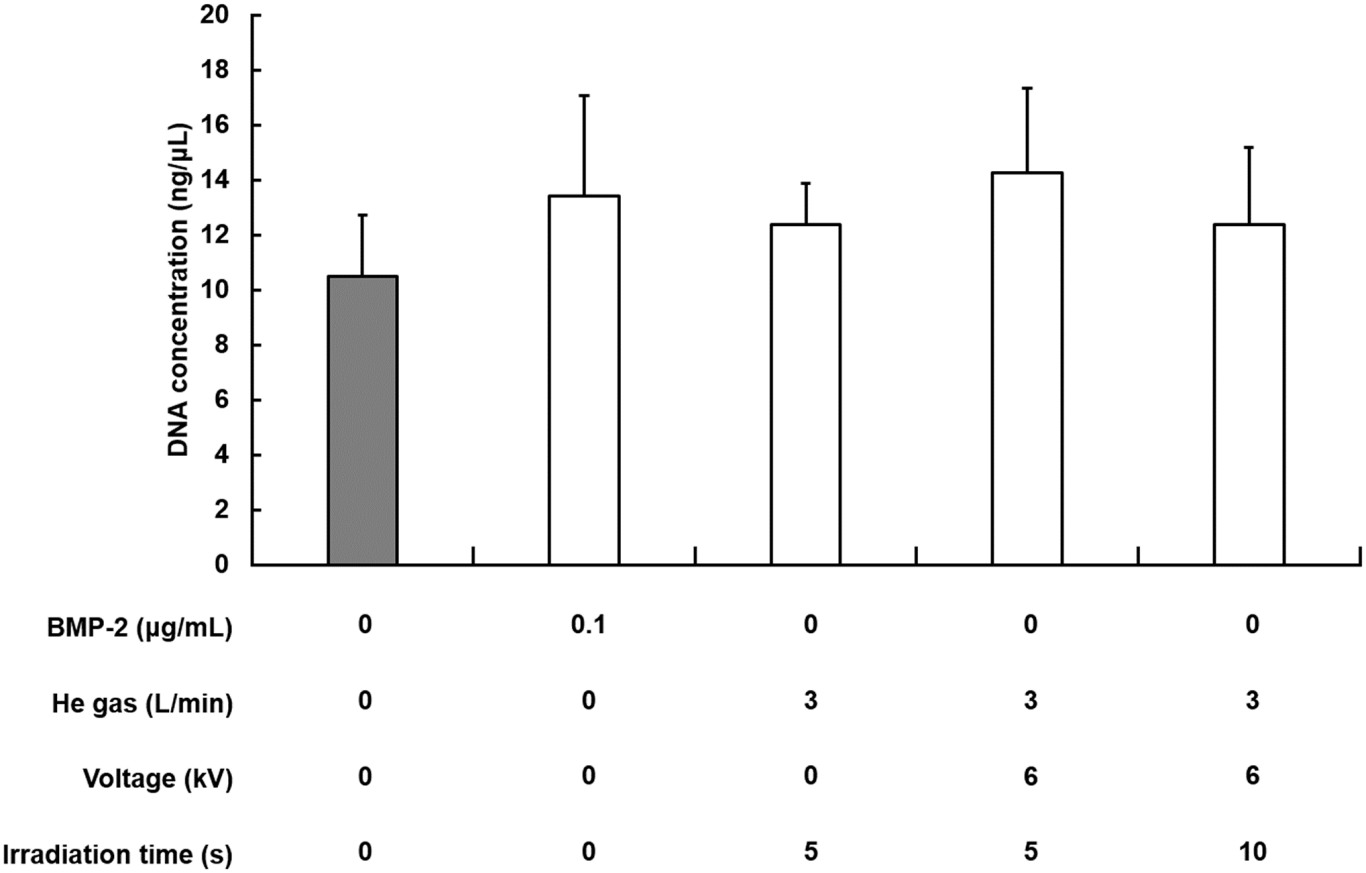
Effect of the plasma irradiation on the proliferation of MC3T3-E1. Cells were incubated for 14 days. DNA was isolated and quantified to evaluate cell proliferation as a percentage of the negative control level (gray). Each value is the mean with SD (*n* = 5). ** *p* < 0.01 vs. control.

The He plasma effect on cell proliferation was found using DNA quantification. Cells incubated with OIR for 14 days were used to ascertain cell proliferation as a control. No significant difference was found among groups in 14 days with or without the 10 repetitions of plasma irradiation (5 s or 10 s each time) (Fig 5).

### 4. Influence of plasma on the calcified deposits

**Fig 6:**
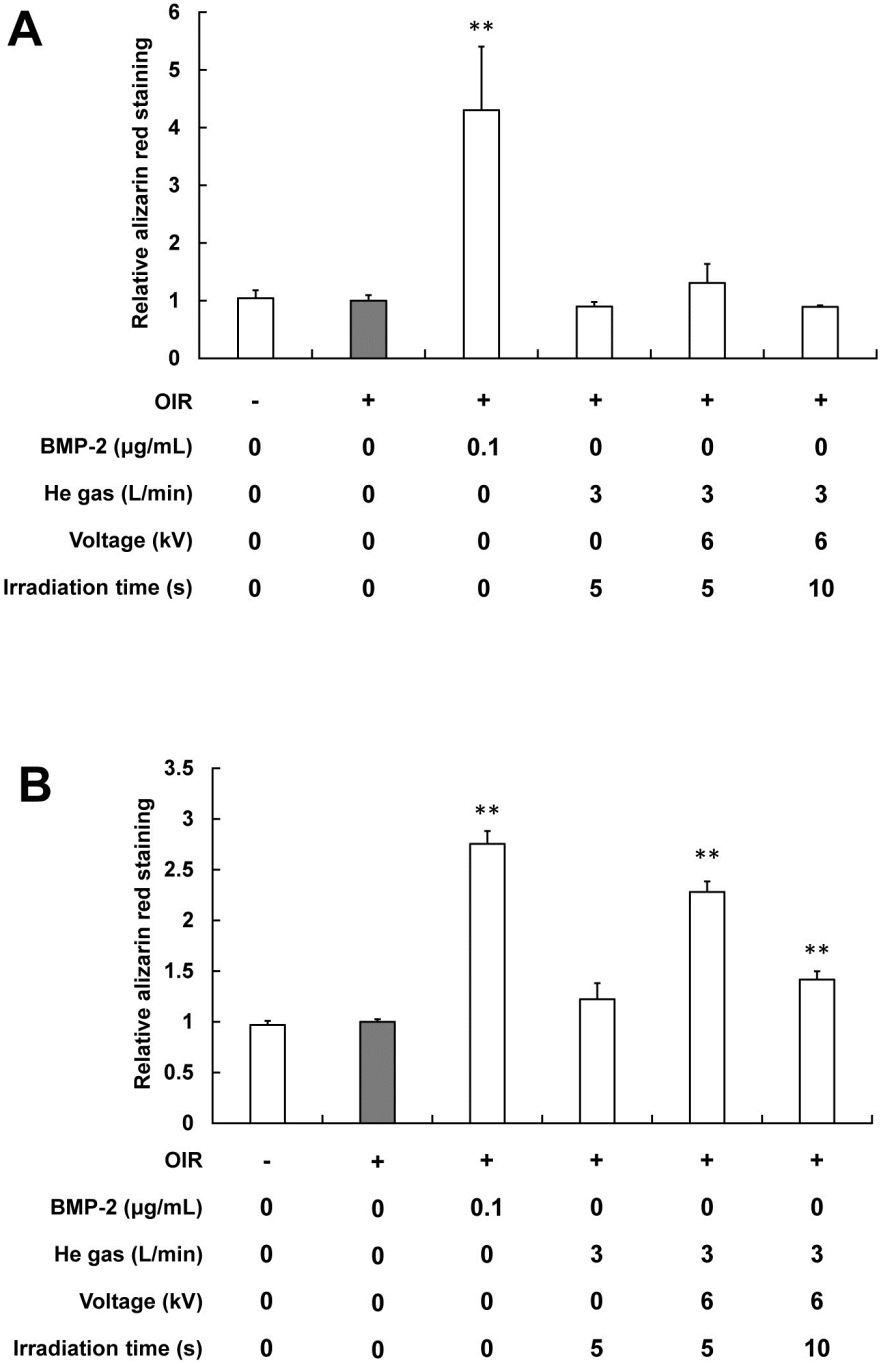
Effect of the plasma irradiation on the mineralization of MC3T3-E1 cells. Cells were incubated for 21 days. Plasma irradiation of cells was conducted in the first week (A) or in the first two weeks (B). Quantification of alizarin red is expressed as a relative value for which the value of the negative control (gray) is set to 1. Each value is the mean with SD (*n* = 5). **p < 0.01 vs. control.

Influences of the plasma on calcified deposits in cells incubated with OIR were determined after 21 days using alizarin red S staining (Fig 6A and 6B). The plasma irradiation of cells was conducted in the first week, during which plasma was irradiated five times (Fig 6A) or the first two weeks, during which plasma was irradiated 10 times (Fig 6B).

Alizarin red S staining of the cells showed a mineralized extracellular matrix as a calcified deposit. In the first two weeks irradiation group, the plasma irradiation for 5 s caused the greatest increase, except for the positive control in alizarin red staining of MC3T3-E1 cells. However, no significant difference was found, except for positive control in the first-week irradiation group.

### 5. ALP and OCN mRNA expressions induced by plasma irradiation

**Fig 7:**
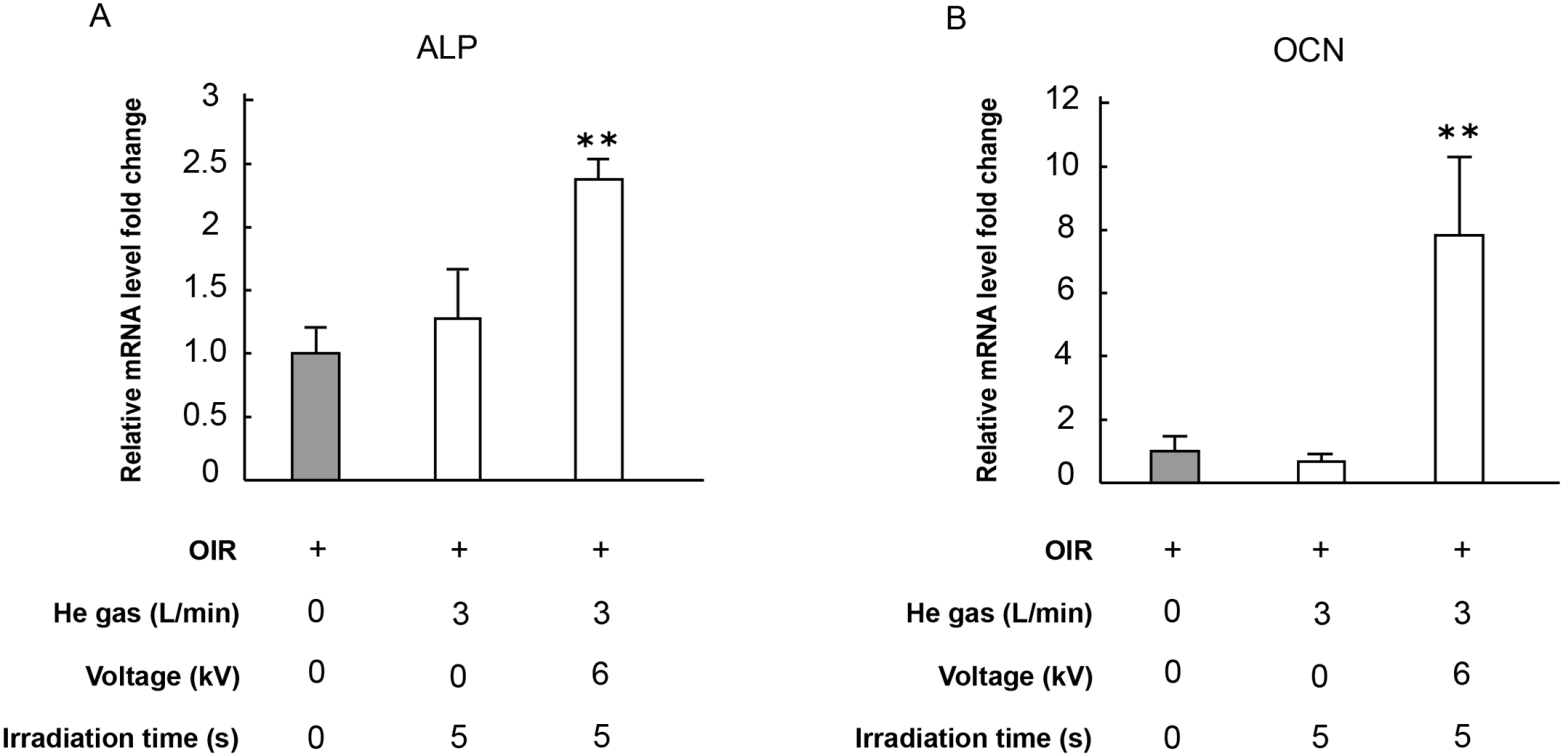
Plasma irradiation effects on MC3T3-E1 mRNA expression. (A) Cells were incubated for 14 days. Plasma irradiation of cells was conducted for two weeks. (B) Cells were incubated for 21 days. Plasma irradiation of cells was conducted for the first two weeks. The mRNA expressions are expressed as a relative value for which the value of the negative control (gray) is set to 1. Each value is the mean with SD (*n* = 5). ** *p*< 0.01 vs. control.

ALP gene is a marker gene expressed increasingly in the early and middle stage of osteoblast differentiation. OCN gene is also a marker gene for which expression increases in the late phase (calcification stage) of osteoblast differentiation following completion of proliferation. As portrayed in Fig 7, ALP and OCN mRNA expressions increased significantly respectively under the same conditions as those shown in Figs 2B and 6B.

### 6. ESR determination of radicals

#### 6. 1 Determination of hydroxyl radical (^•^OH)

**Fig 8:**
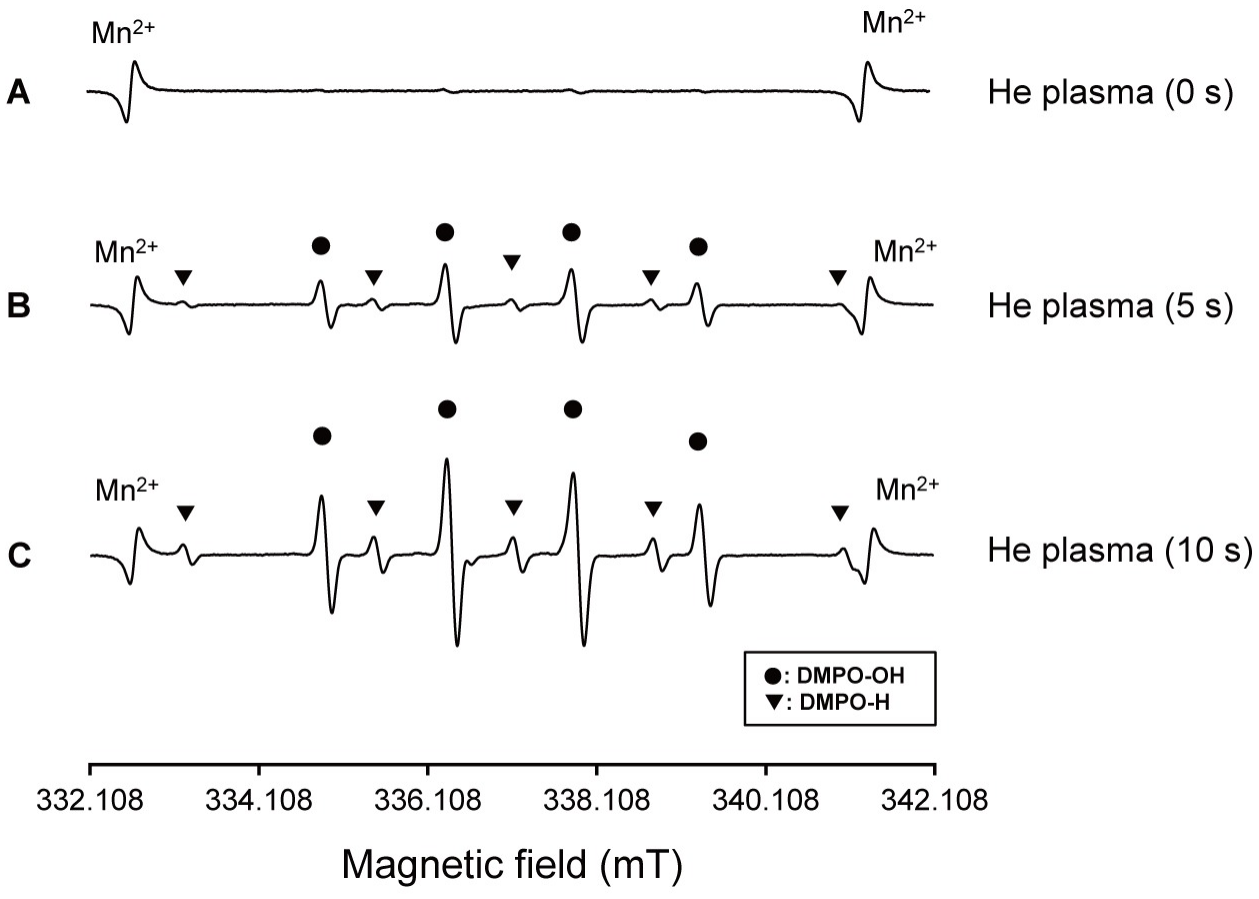
Effects of plasma irradiation for 0 s (a), 5 s (b), and 10 s (C) on ESR spectra of DMPO-OH and DMPO-H adducts obtained from pure water.

When the cell-free pure water with 300 mM DMPO was irradiated with He plasma (6 kV), the ESR signal of DMPO-OH was detected (Fig 8). Fig 8 presents a typical ESR signal after plasma irradiation for 0 s, 5 s, and 10 s. In addition, ^•^H was generated by He plasma. The yield of ^•^OH was increased by He plasma in an irradiation-time-dependent manner.

#### 6. 2 Determination of superoxide anion radicals (^•^O_2_^-^)

**Fig 9:**
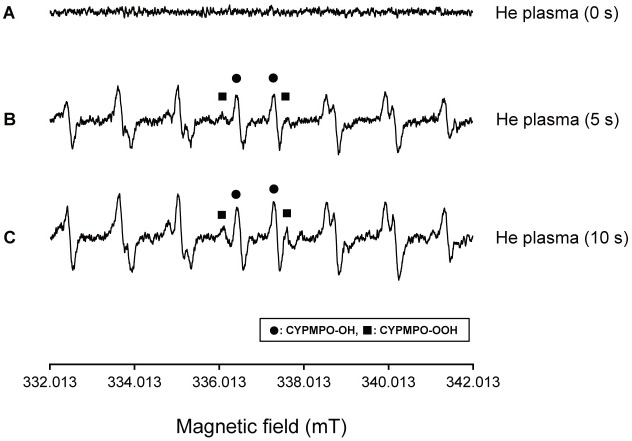
Effects of plasma irradiation for 0 s (a), 5 s (b), and 10 s (C) on ESR spectra of CYPMPO-OOH and CYPMPO-OH adducts obtained from pure water.

Representative spectra of CYPMPO-OH (for ^•^OH determination) and CYPMPO-OOH (for ^•^O_2_^-^ determination) are presented in Fig 9. Results show that He plasma, which was irradiated into pure water (200 μL) with CYPMPO (10 mM), increased both the yield of ^•^O_2_^-^ and ^•^OH in an irradiation-time-dependent manner.

## Discussion

Cold atmospheric plasma produces various biologically active reactive species, particularly ROS including H_2_O_2_, ^•^OH and ^•^O_2_^-^, which are highly reactive radicals or molecules. Various experimental investigations have demonstrated that ROS can affect cell activity, including attenuated proliferation, cell cycle arrest, and increased sensitivity to apoptosis. This study specifically examined short-term effects of plasma irradiation on *in vitro* cell differentiation and the mineralization of osteoblasts (MC3T3- E1 cells) in relation to ROS generation.

To ascertain the effects of the plasma on differentiation, we examined the activity of ALP, an enzyme serving as a marker of osteoblast differentiation [21], in MC3T3-E1 cells treated with the plasma (Fig 2A and 2B). The present study revealed that the plasma irradiation increased the ALP activity of MC3T3-E1 cells, especially for 5 s/day. However, higher activity was found with a greater number of irradiation repetitions. A greater number of irradiation repetitions can be associated with higher activity. Results revealed that appropriate plasma irradiation can promote cell differentiation in MC3T3-E1, which is expected to increase the ALP activity.

We also examined the H_2_O_2_ concentration produced by the plasma because previous reports have described that H_2_O_2_ inhibits osteoblast differentiation [10, 22]. However, no influence of H_2_O_2_ on osteoblastic differentiation was observed in the present study (Fig 4). The discrepancies in the influence of H_2_O_2_ between those described in reports of previous studies and those found in this study are likely to be attributable to the levels of H_2_O_2_ used in the studies. Indeed, 0.2–5.0 mM H_2_O_2_ was used in earlier studies, whereas 6.0 μM H_2_O_2_ was used for this study.

Regarding cell proliferation, no significant difference was found in the cultured MC3T3-E1 cells for any condition of plasma exposure in cultures grown for 14 days (Fig 5). These results indicate that short-term plasma irradiation is not expected to damage osteoblastic cells, suggesting its feasibility for use in clinical applications.

To clarify the plasma effects on bone mineralization further, alizarin red staining was conducted. Calcified deposition occurs in the later period of osteoblastic differentiation [22, 23]. Mineralization occurred in cells treated with the plasma irradiation (5–10 s/time) after 21 days of differentiation (Fig 6A and 6B). In this study, plasma irradiation for 5 s for two weeks caused the greatest increase, except for positive control in alizarin red staining of MC3T3-E1 cells. However, no significant difference was found except for the positive control in the first week irradiation group. Similar to the results of ALP activities, optimum conditions of plasma irradiation for mineralization do exist, such as the period and number of irradiations. Results suggest that cold atmospheric plasma irradiation had an accelerative effect on mineralization on the cell.

This study examined the very short-term effects of continuous plasma irradiation on osteoblastic differentiation. Results show that the plasma irradiation accelerated osteoblastic differentiation. Nevertheless, the underlying mechanisms by which the plasma irradiation accelerates osteoblastic differentiation remain unclear.

Earlier studies recognized ROS as an important factor in various disease processes and biological responses, including cardiovascular diseases, inflammatory conditions, immune disorders, and cancer [20, 24]. Accordingly, in the present, each reactive species generated by cold atmospheric plasma irradiation was detected using ESR spectrometry, which revealed that the yield of radicals was increased in an irradiation-time-dependent manner [25]. Nevertheless, because of the existence of medium and very short life time of radicals, it is unlikely that these radicals affected the cells directly. Rather, chemical species generated secondarily by reaction between the radicals and the culture medium were regarded as stimulating the osteoblastic differentiation of the cells.

Plasma is known to have bactericidal action because of its high concentration of radicals. However, based on results of this study, it is believed to have the capability of accelerating bone regeneration by a low concentration of radicals. Plasma irradiation generates a high concentration of radicals on the tissue surface, and generates a low concentration of radicals and secondarily generated species in the tissue. These features of cold atmospheric plasma, having both bactericidal action and bone regeneration effects, are extremely beneficial for the treatment of chronic infections such as periodontitis. Plasma is also known to exert physical effects such as electromagnetic action in addition to these chemical effects. Therefore, these physical effects must be investigated further. In addition, an important limitation of this study was that a cell line was used to evaluate the effect of cold atmospheric plasma on bone formation. To establish proof of concept for this technology, *in vitro* primary cell studies and *in vivo* model studies should be conducted further.

## Conclusion

Based on results of this study, we concluded that short-term appropriate plasma irradiation can stimulate osteoblast differentiation. We propose that cold atmospheric plasma irradiation might have clinical potential for accelerating periodontitis healing by promoting bone regeneration and bactericidal action. Future work must be undertaken to clarify the biochemical and electromagnetic mechanisms underlying plasma-induced differentiation of MC3T3-E1 cells.
